# Traumatic vs. Non-Traumatic Spinal Cord Injury—Epidemiology, Complications, and Neurological Status During Rehabilitation †

**DOI:** 10.3390/jcm14155209

**Published:** 2025-07-23

**Authors:** Magdalena Mackiewicz-Milewska, Małgorzata Cisowska-Adamiak, Iwona Głowacka-Mrotek, Hanna Mackiewicz-Nartowicz

**Affiliations:** 1 Department of Rehabilitation, Collegium Medicum in Bydgoszcz, Faculty of Health Science, Nicolaus Copernicus University in Toruń, 87-100 Toruń, Poland; malgorzata.cisowska@cm.umk.pl (M.C.-A.); iwona.glowacka@cm.umk.pl (I.G.-M.); 2 Department of Phoniatry and Audiology, Collegium Medicum in Bydgoszcz, Faculty of Medicine, Nicolaus Copernicus University in Toruń, 87-100 Toruń, Poland; hamack@cm.umk.pl

**Keywords:** traumatic spinal cord injury, non-traumatic spinal cord injury, epidemiology, complication

## Abstract

**Background/Objectives**: Spinal cord injuries (SCIs) are among the most debilitating conditions and are a leading cause of disability in young people. This study aimed to analyze the causes of SCIs, assess injury severity using the AIS scale, and evaluate complications during rehabilitation in a hospital setting. **Methods**: The study involved 176 individuals with SCI, including 142 with a traumatic SCI (TSCI) and 34 with a non-traumatic SCI (NTSCI), rehabilitated at various times post-injury. The data on injury causes, paresis type, complications, wheelchair use, gender, age, and treatment methods were collected. The injury severity was assessed using the AIS. **Results**: A significant gender difference was found between the TSCI and NTSCI groups (85.2% male vs. 61.8% male). TSCI individuals were also younger. The causes of TSCI were traffic accidents, falls from height, and diving, while the causes for NTSCI included spinal ischemia, tumors, degenerative disc disease, and inflammation. TSCI individuals had more AIS A lesions (52.8% vs. 26.5%) and more cervical injuries (53.5% vs. 14.7%), whereas NTSCI individuals had more AIS C lesions (38.2% vs. 18.3%) and thoracic damage (58.8% vs. 35.2%). TSCI patients were more often treated surgically (95.7% vs. 61.8%) and used wheelchairs (88% vs. 55.9%). No significant differences were found in terms of complications between the groups, though TSCI individuals underwent more chronic rehabilitation. **Conclusions**: Our research shows that there are significant differences between TSCI and NTSCI both in terms of the level of damage and the severity of damage to neural structures (AIS scales), and thus significant differences in the patients’ functioning in later life for both groups of individuals.

## 1. Introduction

Spinal cord injuries (SCIs) are among the most devastating injuries to the human body. They are also the leading cause of disability in young people [1]. The rate of incidence according to various sources is from 10.4 to 163.4 cases per million per year [2,3]. SCIs can be divided into traumatic (TSCI) and non-traumatic (NTSCI); TSCI is more common, or at least more frequently reported. The data are scarce on the incidence of NTSCI (non-traumatic spinal cord injury) among individuals with SCIs. According to various sources, NTSCI individuals account for 23–61% of the entire SCI group [4,5,6,7,8]. The most common causes of TSCI (traumatic spinal cord injury) are falls from a height, traffic accidents, acts of violence, and diving [1,7,8,9,10]. NTSCI causes are mainly tumors, degenerative disc disease, inflammation, and vascular disease, although some authors include multiple sclerosis, spina bifida, and Arnold–Chiari syndrome in this list [7,9,11]. SCIs cause a very large number of complications depending on many factors, including lesion height, time since injury, and severity of spinal injury. Complications are observed both in the early period, such as motor- and sensory-function disorders, infections, thromboembolic complications, thermoregulation disorders, instability of the circulatory system, and bedsores, and in the late period, such as joint contractures, osteoporosis, and urinary tract infection [5,6,7,12,13].

The aim of the study was to analyze traumatic and non-traumatic causes of SCIs, assessing the severity of the injury on the AIS scale and complications occurring in individuals with SCI during rehabilitation in a hospital setting.

## 2. Materials and Methods

This retrospective study included 176 individuals with SCIs who were rehabilitated at various times after the injury. The study was conducted at the Rehabilitation Department of the University Hospital No. 1 in Bydgoszcz in the period 2009–2021. The study was approved by the Bioethics Committee of Nicolaus Copernicus University in Toruń, Collegium Medicum in Bydgoszcz (consent number KB 567/2022; approval date 13 December 2022). Individuals were admitted either directly from the acute phase centers for early rehabilitation (usually up to 3 months after the injury), or later; so-called late rehabilitation was considered to be over 3 months from the injury, although it could be many months or even years later. The following variables were assessed: the time from injury; type of paresis; cause of the injury; presence of complications, such as heterotopic ossification (HO), pressure ulcers, infections, deep vein thrombosis (DVT), pulmonary embolism (PE), hematoma of muscle during rehabilitation, and fractures during rehabilitation; use of a wheelchair; sex and age; and the treatment method, operational or conservative. In addition, the severity of the SCIs was assessed on the AIS scale and classed as A, B, or C. The D scale was not diagnosed in any patient. Individuals who were repeatedly hospitalized in the Rehabilitation Department after their SCI were analyzed only once during the first stay, as early as possible after the injury. The individuals were divided into two groups: individuals with TSCI and individuals with NTSCI. Characteristics of individuals with TSCI and NTSCI are presented in Table 1. Only individuals with acute onset NTSCI were included in the study.

The inclusion and exclusion criteria were as follows.

Inclusion Criteria:

- Age > 18 years
- Individuals with spinal cord injury (SCI), both traumatic (TSCI) and non-traumatic (NTSCI).
- Only individuals with acute onset of NTSCI.

In our study, “acute onset NTSCI” was defined as a sudden neurological impairment resulting from a non-traumatic cause, characterized by a rapid progression of symptoms leading to spinal cord dysfunction within a short time frame prior to hospital admission. Specifically, patients were included if they experienced a clear and sudden clinical onset of spinal cord injury symptoms (e.g., paresis, sensory deficits), typically developing over hours to days, rather than a gradual or chronic progression. The group included patients rehabilitated at the Rehabilitation Department of University Hospital No. 1 in Bydgoszcz during the period 2009–2021. Individuals were hospitalized for the first time after the injury, either in early rehabilitation (up to 3 months post-injury) or late rehabilitation (more than 3 months post-injury). Individuals all had SCI severity assessed as AIS grades A–D.

Exclusion Criteria:

- Patients with multiple hospitalizations—only the first hospitalization after a SCI was analyzed.
- No neurological impairment after the SCIs.
- Individuals with NTSCI of chronic or non-acute onset.
- Patients with incomplete data on injury timing, injury type, neurological status, or rehabilitation course.

For most parameters, the data represent the number of individuals (n), including the percentage of total number (in parentheses). For age and time since injury, medians and quartiles are shown (median [Q1, Q3]). *p*-values are given for the difference between the TSCI group and NTSCI group.

## 3. Statistics

All analyses were performed assuming a significance level of 0.05 in advance. For the cross tables of size 2 × 2, Fisher’s exact test was used to examine the relationship between the variables. When more groups were included, the Chi-square test of independence was used if all expected frequencies for subsamples were greater than five. In all other cases, the exact Fisher–Freeman–Halton test was used. Continuous variables (age and time between injury and examination) were verified for the existence of outliers using boxplots and normality in subgroups using the Shapiro–Wilk test. As a result, Student’s *t*-test was used to compare mean age between groups, whereas the Mann–Whitney U test compared medians for the time between injury and examination.

## 4. Results

**Figure 1:**
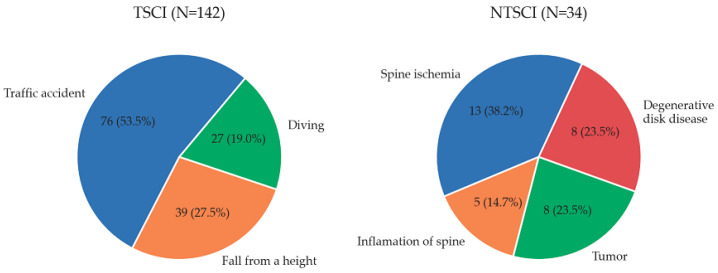
Characteristics of the causes of SCIs.

Among the analyzed individuals, there were 142 individuals with TSCI (80.7%) and 34 NTSCI individuals (19.3%). There was a statistically significant difference in the sex of individuals admitted for TSCI compared with NTSCI (85.2% M vs. 61.8%): *p* = 0.0035. The individuals with trauma were younger (34.61 ± 13.79) than the non-traumatic group (54.06 ± 15.94) *p* < 0.001 (Table 1). TSCI causes are as follows, in order from most observed to least observed: traffic accident (53.5%), fall from height (27.5%), and diving (19%). Causes of sudden onset acquired NTSCI are spinal ischemia (38.2%), tumor (23.5%), degenerative disc disease (23.5%), and inflammation of spine (14.7%) (Figure 1). Individuals with TSCI had significantly more type A lesions on the AIS scale than NTSCI individuals, 75 (52.8%) vs. 9 (26.5%), *p* 0.0071, while the AIS type C lesion was more common in NTSCI individuals: 13 (38.2%) vs. 26 (18.3%), *p* 0.0199. In individuals with trauma, damage to the C segment was more frequent (53.5% vs. 14.7%), *p* < 0.001, while in individuals with NTSCI, damage to the thoracic segment was more common, 20 (58.8%) vs. 50 (35.2%), and the lumbar segment, 9 (26.5%) vs. 16 (11.3%); these were statistically significant differences. Individuals presenting as a result of TSCI were more likely to be treated with surgery (95.7% vs. 61.8%), less likely to be treated conservatively (4.2% vs. 38.2%), and more likely to use a wheelchair (88% vs. 55.9%) (Table 1). The median time from injury to hospitalization for TSCI individuals was 6 months and for NTSCI individuals was 1.5 months.

No statistically significant differences were found between the TSCI and NTSCI groups in the incidence of the analyzed complications, such as DVT, muscle hematoma, bone fractures, HO (heterotopic ossification), PE (pulmonary embolism), decubitis ulcers, urinary tract infections, respiratory tract infections, and autonomic dysreflexia (Table 2).

**Figure 2:**
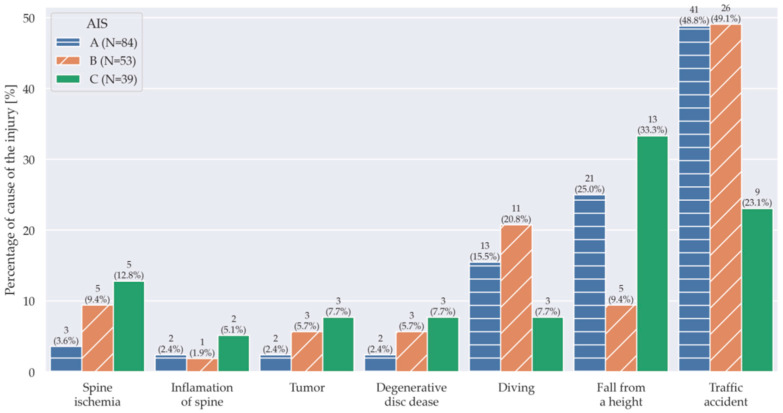
The severity of neurological disorders (AIS scale) depends on the cause of the injury.

When analyzing the severity of damage on the AIS scale, individuals with TSCI had a severe degree of damage, indicated by AIS A, more frequently than individuals with NTSCI, regardless of the cause. It should be noted that individuals after diving had AIS A and B in as many as 89% of cases, and individuals after traffic accidents had both in 88.1% of cases. Individuals with NTSCI were mainly in the ASIA C and B group, except for individuals with myelitis, who had the same rate of damage type A and C. (Figure 2).

For all parameters, the data represent the number of individuals (n) including the percentage of total number (in parenthesis) with respect to the degree of impairment in AIS A, B, C.

## 5. Discussion

In our study, TSCI individuals accounted for 80.7% and NTSCI individuals for 19.3% of hospitalized individuals. The smaller number of NTSCI individuals undergoing rehabilitation may be due to the fact that people with smaller neurological impairment—more individuals in the AIS C group and with an injury at the L-S level—use outpatient rehabilitation more often. Moreover, we did not include patients without neurological deficits, either in the TSCI or NTSCI groups, whereas such criteria are not always considered in studies on the epidemiology of SCIs. In the study conducted by Ge, which included 756 patients with SCIs (both TSCI and NTSCI), as many as 61.6% were individuals with NTSCI [7].

On the other hand, in a study on a group of 169 individuals also undergoing rehabilitation, Cosar [8] described the proportion of individuals with TSCI to NTSCI as 77% to 23%, which was similar to our findings. In the present study, TSCI individuals were statistically significantly younger than NTSCI individuals, with a mean age of 34.61 vs. 54.06, and were predominantly male: 85.2% vs. 61.8% in the NTSCI group. Epidemiological data for individuals with TSCI are quite numerous and not all are consistent with our research. According to the National Spinal Cord Injury Statistical Center, Birmingham, Alabama [14], the age of TSCI individuals has increased in recent decades from 28.7 to 41 years, which is associated with an aging population. Research conducted by others confirmed these findings [3,15]. Kang et al. [3] reported that the average age in developed countries is 14.6 to 67.6 and in undeveloped countries 29.5 to 46. The male to female ratio varies from 1.10:1 to 6.69:1 depending on the country. Güzelküçük et al. [16] reported that the TSCI male-to-female ratio is 4:1 and the mean age is 34.6, which is consistent with our research. Cosar et al. [8] reported in their studies that the average age of individuals with TSCI is 37.8, and the ratio of men to women is 67.6% vs. 32.3%. In our study, NTSCI individuals were significantly older—the mean age was 54.06, and the male to female ratio was 61.8% vs. 39.2%, which is consistent with studies by other authors [8,17]. Similar results to ours were also obtained by Khadur et al. in a retrospective study involving 649 patients rehabilitated in the Rehabilitation Department in Wuhan; the mean age of patients with TSCI was 35.4, and for those with NTSCI it was 54.8 [10]. Similarly, in Scivoletto’s [18] work, the age of individuals with NTSCI is described as older than those with TSCI, and the ratio of men to women in NTSCI is 55% to 45%, and in TSCI 80% to 20%, which is consistent with our research. In addition, McKinley [6], in his study on a group of 220 individuals with TSCI and NTSCI, observed that there were definitely fewer men in the NTSCI group, at 50%, while in the TSC group, the proportion was 84%.

The older age of patients with NTSCI may primarily result from the causes of the SCIs, such as degenerative disc disease or tumors, which are often associated with more advanced age [19,20]. On the other hand, patients with TSCI tend to be younger, which can be explained by a greater propensity for risk-taking and recklessness at this age, especially among young men—factors that are common causes of car accidents [20].

In our analysis, the most common cause of TSCI was a traffic accident (53.5%), followed by a fall from a height (27.5%), and diving (19%). In the NTSCI group, the causes were ischemia (38.2%), disc degenerative disease (23.5%), tumor (23.5%), and inflammation (14.7%). The causes of TSCI and NTSCI are consistent with studies by other authors [8,21,22,23]. According to Khadour [10], the main cause of NTSCI is tumors (34.5%), while according to Choi Y [19], it is degenerative disc diseases, and the proportion continues to increase with an aging population. Some authors cite falling from a height as the first cause of TSCI [23]. They also point out that gunshot wounds are often mentioned as a cause of an SCI injury. In the Polish population, this cause is completely irrelevant. In the literature, it is stated that gunshots are the cause of 2.4% to 17% TSCIs [8,24,25]. In the first author of this manuscript’s 25 years of practice at the Rehabilitation Department, so far, only one patient with a SCI has been hospitalized as a result of gunshots.

One of the causes of TSCI is diving (especially jumping headfirst into a pool or lake). In our research, it was the cause of TSCI in 19% of cases. Similar data for the Polish population were presented by Tederko et al. [26]. This is a relatively high percentage of cases, despite extensive education among the public, especially young people. In this group of individuals, there are many young people, including those under 20 years of age. This kind of accident occurs mostly in the summer. Injuries mainly affect the cervical spine and often result in an AIS A lesion that consequently leads to a long-term and severe disability [27].

It should be emphasized that, in Poland, there is no national database of individuals with TSCI and NTSCI [28], which is also noted by New et al. [29], who stated that there is no database of these individuals in Eastern Europe. It is therefore difficult to describe the exact data on the epidemiology of this group of individuals in Polish society. However, according to Guan et al., the number of new cases of SCIs has been decreasing over the last 30 years in eastern and central European countries [22].

Among the causes of NTSCI, some authors also include congenital defects such as spinal dysraphism, Chiari malformations, skeletal malformations or genetic disorders (hereditary spastic paraparesis, spinocerebellar ataxias, adreno-myeloneuropathy, leukodystrophies), and spinal muscular atrophy, as well as multiple sclerosis [11,30]. Only individuals with acute onset paresis due to acquired NTSCI were included in our study. In the present study, the most common non-traumatic cause of SCIs was vascular disease (ischemia), 38.2%, followed by tumor and degenerative disc disease, 23.5%, and the least common was inflammation, 14.7%. In studies conducted by Scivoletto [18], the causes of NTSCI are degenerative disease of the spine with spinal cord involvement (32%), and vascular (24%), inflammatory (23%), and neoplastic diseases (21%), which is consistent with our research. In their work, New et al. [31] argue that in developed countries, degenerative disc disease and tumors of the spinal canal are dominant as the cause of NTSCI, while in developing countries the main cause of NTSCI is infections.

Among the individuals we analyzed, people after TSCI were much more likely to use a wheelchair and were subjected to the so-called late rehabilitation more often than individuals with NTSCI. TSCI individuals comprised 93%; they were admitted more than 3 months after the injury. The median time from injury to hospitalization in the Rehabilitation Department was 6 months for TSCI individuals and 1.5 months for NTSCI individuals. This does not mean that individuals with TSCI did not benefit from early rehabilitation, but they were also admitted to the so-called late rehabilitation. In Poland, according to the guidelines of the main state insurer, individuals with neurological dysfunctions, e.g., under SCIs, can use hospital rehabilitation once a year after undergoing so-called early rehabilitation, lasting a maximum of about four months, with the so-called chronic rehabilitation lasting up to six weeks. In our opinion, a high degree of disability, as evidenced by, among other things, the need to use a wheelchair, is the reason for a more frequent use of hospital rehabilitation in the late period after an injury.

The individuals we analyzed in the TSCI and NTSCI groups differed in the severity of damage assessed in the AIS and in the level of damage. Individuals with TSCI had more AIS A lesions and NTSCI individuals had more AIS C lesions, which was statistically significant in both cases. Similarly, Gedde [5] observed AIS A individuals more often among TSCI individuals than NTSCI individuals, and among NTSCI individuals, there were more AIS C individuals than in the TSCI group, although the proportions differed from our study. On the other hand, Halvorsen [32] most often qualified individuals with NTSCI as AIS D. Among the individuals with spinal cord damage hospitalized in our department, individuals with AIS D were not observed. In Poland, people with that kind of injury outcome were mostly rehabilitated in outpatient clinics.

Our research shows that the incidence of complications such as infections, thromboembolic complications, autonomic dysreflexia, decubitus ulcers, HO, intramuscular hematomas or fractures during rehabilitation did not differ statistically significantly in the TSCI and NTSCI groups, although bone fractures due to osteoporosis during rehabilitation and symptoms of dysreflexia or respiratory infections were observed only in the group of individuals with TSCI; however, these differences were not statistically significant, perhaps due to the small group of individuals with these complications. However, when the individuals admitted in the early and late rehabilitation group were analyzed, complications such as DVT or intramuscular hematomas occurred statistically significantly more often in the early rehabilitation group. The incidence of DVT in the early rehabilitation group (both TSCI and NTSCI) was 22.9% vs. 4.7% in the late rehabilitation group, which is consistent with our previous studies [33]. In a previous study published by the authors, the incidence of DVT in individuals with SCIs up to three months after injury was 22% [33]. Chung et al. [34] reported that the risk of DVT and PE in individuals with SCIs is 16.9 and 3.6 times higher within 3 months of the injury than in the general population. In Shang’s meta-analysis of 223,221 SCI patients, the incidence of DVT was 9.3%, with 10.9% in the acute phase and 5.3% in the chronic phase of a SCI [13].

In our study, the overall incidence of DVT, without division into acute and chronic phases, was 12% in TSCI and 11.8% in NTSCI. The higher value in our study may be due to the fact that most of our patients underwent DVT screening by Doppler ultrasonography of the lower limb veins, which is standard practice in our Rehabilitation Department.

Intramuscular hematomas are a relatively rare complication; however, in our group of individuals, their incidence was 2.8% (TSCI) vs. 5.9% (NTSCI). It should be emphasized that all individuals with intramuscular hematomas received low-molecular-weight heparin (LMWH) in a prophylactic or therapeutic dose or oral anticoagulants. This is an often overlooked aspect of prophylaxis or anticoagulant therapy. Intramuscular hematomas were localized in the muscles of the lower limbs or the hip girdle. However, these complications should always be borne in mind in the event of sudden swelling of a limb or a sudden decrease in hemoglobin [35]. Yeung [36] also described three cases of hematomas in SCI individuals, all of whom received anticoagulation therapy. A smaller number of intramuscular hematocytes in the chronic group should be associated with less frequent use of antithrombotic prophylaxis long time after the injury and less frequent thromboembolic complications requiring the use of anticoagulation.

## 6. Conclusions

Our research shows that there are significant differences between TSCI and NTSCI both in terms of the level of damage and the severity of damage to neural structures (AIS scales), and thus significant differences in the patients’ functioning in later life for both groups of individuals. Traumatic causes of SCIs mainly affect the cervical region and cause greater disability, associated with the need to move around in a wheelchair. So-called long-term hospital rehabilitation after injury is mainly used by individuals with a greater degree of disability. These conclusions should be used in planning and providing appropriately targeted social care for both TSCI and NTSCI individuals. It is necessary to conduct epidemiological studies of individuals with TSCI and NTSCI across the whole of Poland. This study highlights key differences between patients with traumatic and non-traumatic spinal cord injuries during rehabilitation, emphasizing the need for individualized rehabilitation strategies. The findings support early referral to rehabilitation, especially for NTSCI patients, and underline the importance of systematic screening for complications such as pressure ulcers or thromboembolic events. A better understanding of neurological status and typical complication profiles in each group can help optimize clinical decision-making, goal-setting, and resource allocation in rehabilitation settings.

## 7. Limitations of the Study

One of the limitations of the study is the heterogeneous group of individuals; it included individuals both in the early rehabilitation period, i.e., up to 3 months, and in the late period, even several years after the injury, which could have influenced the occurrence of complications. In addition, the study did not take into account neuropathic pain as a complication due to the inclusion of analgesic treatment in some individuals before the start of hospital rehabilitation. At the beginning of hospital rehabilitation, they did not report any pain.

Other limitations of the study are the retrospective nature of the study and the limitations resulting from the single-center study design.

Another limitation of this study is the unequal group sizes, with 142 patients in the traumatic SCI (TSCI) group compared to only 34 patients in the non-traumatic SCI (NTSCI). This imbalance may have introduced potential confounding effects and affected the statistical power of between-group comparisons. A smaller sample size in the NTSCI group reduces the ability to detect significant differences and increases the risk of Type II errors. Additionally, the difference in group sizes might have influenced the variability and generalizability of the results.

## Figures and Tables

**Table 1:** Individual demographic and clinical characteristics.

	TSCI (N = 142)	NTSCI (N = 34)	*p*-Value
Gender: male (M)	121 (85.2%)	21 (61.8%)	0.0035
Age (Mean (SD))	34.61 (13.79)	54.06 (15.94)	<0.001
Spastic paraparesis (n, %)	43 (30.3%)	16 (47.1%)	0.0711
Flaccid paraparesis (n, %)	23 (16.2%)	16 (47.1%)	<0.001
Tetraparesis (n, %)	75 (52.8%)	3 (8.8%)	<0.001
Time since injury (months) (median [Q1, Q3])	6 [2.13; 16.8]	1.5 [1; 2]	<0.001
AIS A (n, %)	75 (52.8%)	9 (26.5%)	0.0071
AIS B (n, %)	41 (28.9%)	12 (35.3%)	0.5330
AIS C (n, %)	26 (18.3%)	13 (38.2%)	0.0199
Cervical spine injury (n, %)	76 (53.5%)	5 (14.7%)	<0.001
Thoracic spine injury (n, %)	50 (35.2%)	20 (58.8%)	0.0183
Lumbosacral spine injury (n, %)	16 (11.3%)	9 (26.5%)	0.0302
Surgery for SCI	136 (95.7%)	21 (61.8%)	<0.001
Conservative treatment SCI	6 (4.2%)	13 (38.2%)	<0.001
Wheelchair	125 (88.0%)	19 (55.9%)	<0.001

Abbreviations: AIS—The American Spinal Injury Association Impairment Scale; TSCI—traumatic spinal cord injury; NTSCI—non-traumatic spinal cord injury; SCI—spinal cord injury; AIS A—impairment complete according to AIS; AIS B and C—impairment incomplete according to AIS.

**Table 2:** Complications during the rehabilitation stage.

	TSCI (n = 142)	NTSCI (n = 34)	*p*-Value
DVT (n)	17 (12%)	4 (11.8%)	0.8496
Muscle hematoma (n)	4 (2.8%)	2 (5.9%)	0.2912
Bone fractures (n)	3 (2.1%)	0 (0%)	0.6273
HO (n)	23 (16.2%)	2 (5.9%)	0.1338
PE (n)	1 (0.7%)	1 (2.9%)	0.1977
Decubitus ulcers (n)	12 (8.5%)	3 (8.8%)	0.7678
Urinary tract infection (n)	64 (45.1%)	15 (44.1%)	0.9270
Respiratory tract infection (n)	11 (7.7%)	0 (0%)	0.1091
Autonomic dysreflexia (n)	11 (7.7%)	0 (0%)	0.1091

Abbreviations: *p*-values are given for the differences between the TSCI group and NTSCI group. DVT—deep vein thrombosis; HO—heterotopic ossification; PE—pulmonary embolism. For all parameters, the data represent the number of individuals (n) including the percentage of total number (in parentheses).

## Data Availability

The authors will make the raw data supporting this article’s conclusions available upon request.
